# Morphological distribution of μ chains and cd15 receptors in colorectal polyp and adenocarcinoma specimens

**DOI:** 10.1186/1472-6890-13-8

**Published:** 2013-03-01

**Authors:** Caterina Defendenti, Fabiola Atzeni, Anna Maria Croce, Elena Mussani, Simone Saibeni, Simona Bollani, Silvia Grosso, Piero Luigi Almasio, Savino Bruno, Piercarlo Sarzi-Puttini

**Affiliations:** 1 Laboratory Unit and Divisions of Fatebenefratelli Hospital, Milan, Italy; 2 Rheumatology Unit, L. Sacco University Hospital, Milan, Italy; 3 Pathology, Fatebenefratelli Hospital, Milan, Italy; 4 Gastroenterology, Fatebenefratelli Hospital, Milan, Italy; 5 GI & Liver Unit, DIBIMIS, Policlinico, University of Palermo, Palermo, Italy; 6 Unità di Sierologia e Autoimmunità, Ospedale Fatebenefratelli, Corso di Porta Nuova 23, Milan, 20133, Italy

**Keywords:** Inflammatory bowel disease, B-1 cells, Sialyl-LewisX, Colorectal adenocarcinoma, Matrix metalloproteinases

## Abstract

**Background:**

We have recently investigated the localisation of immunoglobulin-producing cells (IPCs) in inflamed intestinal tissue samples from patients with inflammatory bowel disease (IBD), and identified two main patterns of B lymphocyte infiltration: one characterised by the moderate strong stromal localisation of small B1 cell-like IgM+/CD79+/CD20-/CD21-/CD23-/CD5 ± IPCs, and the other by the peri-glandular localisation of IPCs with irregular nuclei that had surface markers specific for a B cell subset (IgM and CD79), but quantitative differences in their λ and κ chains. The same patients were also tested for CD15+ receptors, which were localised on inflammatory cell surfaces or in the crypts of the intestinal epithelium. CD15+ receptor distribution in inflamed tissues was limited to the cell structures. The aim of the study was to analyse variations in IPCs and CD15+ cell morphology or distribution in bowel biopsy specimens taken from patients with pre-malignant polyps or adenocarcinomas.

**Methods:**

IPCs were analysed by means of immunofluorescence using polyclonal goat anti-human μ chains. The pre-malignant polyp specimens were tested for B cell surface phenotype λ and κ chains, CD79, CD20, CD21 and CD23 using an immunoperoxidase method. CD15+ cells were evaluated using the immunoperoxidase method and monoclonal anti-CD15 IgM.

**Results:**

The study involved 14 patients (four with pre-malignant polyps and 10 with colorectal adenocarcinomas). The distribution of μ chains and CD15 markers varied in all of the biopsies, but delineated normal cell structures in the pre-malignant polyp specimens. B cell surface phenotype analysis of μ chain-positive cells identified a subset of CD79+/CD20-/CD21-/CD23- IPCs. The IPCs in certain areas showed the sporadic disintegration of inflammatory cell membranes or the accumulation of fluorescence in individual cells. IPC membrane disintegration was particularly marked in all of the adenocarcinoma samples, in which the CD15 markers also showed epithelial cell involvement. Furthermore, six of the ten adenocarcinoma samples had atypical and reorganised membranes that expressed an excess of both receptors and isolated small portions of tissue within the tumour.

**Conclusion:**

The findings of this preliminary morphological study suggest the presence of membrane disintegration and remodelling mechanisms in the tumours. The newly-formed membranes expressed high concentrations of inflammatory cell receptors that can confer adhesive properties.

## Background

A number of studies have shown that the origin of cancer may lie in chronic inflammation, and that inflammation is a critical component of tumour progression [1]. De Visser *et al*. [2] have shown that B lymphocytes play a critical role in initiating chronic inflammation during pre-malignancy, thus supporting the idea that, although important, oncogene expression in “initiated” cells is not solely responsible for malignant progression. The B1 cell subset of B lymphocytes, which are mainly present in the pleural and peritoneal cavities, can migrate to inflammatory sites and differentiate into mononuclear phagocytes with macrophage-like phenotypes [3]. They also secrete numerous antibodies against common membrane phospholipids, such as phosphatidylcholine [4]. Various authors have shown a physical interaction between the B16 murine melanoma cell line and B1 cells in experimental co-cultures [5,6]. The same authors evaluated the interaction between B1 and tumour cells in order to investigate whether this gives rise to signals that influence melanoma cell metastases, and observed cell-to-cell interactions due to adhesive molecules expressed on the surfaces of both types of cells. The expression of a high-molecular-weight mucin-like sialoglycoprotein positively correlates with progression to metastatic phenotypes. Electrophoretic separation of tumour tissue extract on 3% polyacrylamide gel followed by direct staining with the FH6 monoclonal antibody has shown that the glycoproteins of the highest molecular weight contained sialyl-LewisX, the CD15 antigen, and mucins. Selectins are adhesive receptors that normally recognise some vascular mucin-type glycoproteins bearing the carbohydrate sialyl-LewisX structure, and facilitate leukocyte rolling in blood vessels. P-selectin deficiency reduces tumour growth and metastases in mice treated with a receptor antagonist peptide [7]. We have recently investigated the localisation of immunoglobulin-producing cells (IPCs) in inflamed intestinal tissue samples from 96 patients with inflammatory bowel disease (IBD: 64 with ulcerative colitis [UC] and 32 with Crohn’s disease [CD]), and identified two main patterns of B lymphocyte infiltration. One (42.7% of the cases) was characterised by the moderate-strong stromal localisation of small B1 cell-like IgM+/CD79+/CD20-/CD21-/CD23-/CD5 ± IPCs [8], and the other (35.4% of the cases) by the peri-glandular localisation of IPCs with irregular nuclei that had surface markers specific for a B cell subset (IgM and CD79), but quantitative differences in their λ and κ chains. The same 96 patients were also tested for CD15+ receptors, which were localised on inflammatory cell surfaces or in the crypts of the intestinal epithelium [9]. The aim of this study was to evaluate the distribution of μ chains and CD15+ receptors in biopsy specimens of pre-malignant polyps and adenocarcinomas in order to identify marker distribution in a tumoral context.

## Methods

### Patients

Four intestinal pre-malignant polyps and ten colorectal adenocarcinoma tissue samples were taken from patients undergoing complete colonoscopy at Fatebenefratelli Hospital in Milan, Italy, all of whom signed an informed consent form in accordance with the Declaration of Helsinki. The study was approved by the Ethics Committee of Fatebenefratelli Hospital. The diagnoses were confirmed by means of standard endoscopic and histological criteria (additional hematoxylin and eosin staining). Six sections of each biopsy specimen were taken from premalignant and neoplastic foci and analysed. Control specimens were taken from ten patients with normal endoscopic results, and without any macroscopic signs of inflammatory or neoplastic disease.

### Immunofluorescence method

The intestinal sections (3 μm) were deparaffined (72°C) and pre-treated to enhance antigenicity (95°C × 36 min), and the tissue distribution of the μ chains was investigated using indirect immunofluorescence (IIF). The sections were incubated with horse serum for 30 minutes at room temperature in order to prevent non-specific binding. After removing the blocking solution, the sections were incubated with a goat anti-human IgM diluted 1:100 (μ chain-specific, Vector Laboratories, Burlingame, California, USA, code FI-3020 ). While being protected from exposure to direct light at 37°C for one hour, the samples were washed four times for five minutes in high salt PBS (NaCL 4 M and PO4 buffer) and mounted. Images of four randomly selected areas of each sample were recorded at 20× and 40× magnification (Eurostar Instruments, Lubeck, Germany).

### Immunoperoxidase method

The pre-malignant polyp and colorectal adenocarcinoma specimens were deparaffined (72°C), and pre-treated to enhance antigenicity at a specific temperature and/or time of incubation. Immunoperoxidase was used to detect the distribution of the tissue expression of CD15. The premalignant polyp sections (3 μm) were also used to detect λ and κ chains, CD79, CD20, CD21 and CD23. The murine monoclonal antibody (mAb) λ and κ chains, CD15, CD79, CD20, CD21 and CD23 (Medical System, Tucson, Arizona, USA) were applied to formalin fixed, paraffin-embedded tissue using a Ventana automated slide stainer (Medical System), after which the biotinylated secondary antibodies were added, followed by the streptavidin-horseradish peroxidase conjugate (the anti-CD was diluted for use with Ventana detection kits and automated slide stainers). Each step in the staining protocol included incubation for a precise period of time at a specific temperature. At the end of each incubation step, the sections were rinsed by means of the Ventana automated slide stainer in order to halt the reaction and remove any unbound material that would hinder the desired reactions in subsequent steps. A coverslip solution was used in the slide stainer in order to minimise the evaporation of the aqueous reagents from the specimen-containing slide. The complex was then visualised using a hydrogen peroxide substrate and 3, 3’-diaminobenzidine tetrahydrochloride (DAB) chromogen.

## Results

### Study population

The demographic and histological characteristics of the 14 fourteen patients are shown in Table 1.

**Table 1:** Demographic and hystological characteristics of the patients under study

	**Pre-malignant polyps**	**Adenocarcinomas**
**Patients**	4	10
**Male/female ratio**	2/2	7/3
**Age at diagnosis (mean, range)**	69 ± 4	76 ±7
**Localisation**		
**Colon/Rectum-sigma**	0/4	4/6
**IPC-positive cells**		
**B1-like cells /irregular nuclei**	4/0	6/4
**Dysplasia**		
**Mild**	4 (100%)	2 (20%)
**Moderate**		4 (40%)
**Severe**		4 (40%)

### Fluorescent anti-μ chain marker localisation in pre-malignant polyps identified B cells with a low cytoplasmic/nuclear ratio

**Figure 1:**
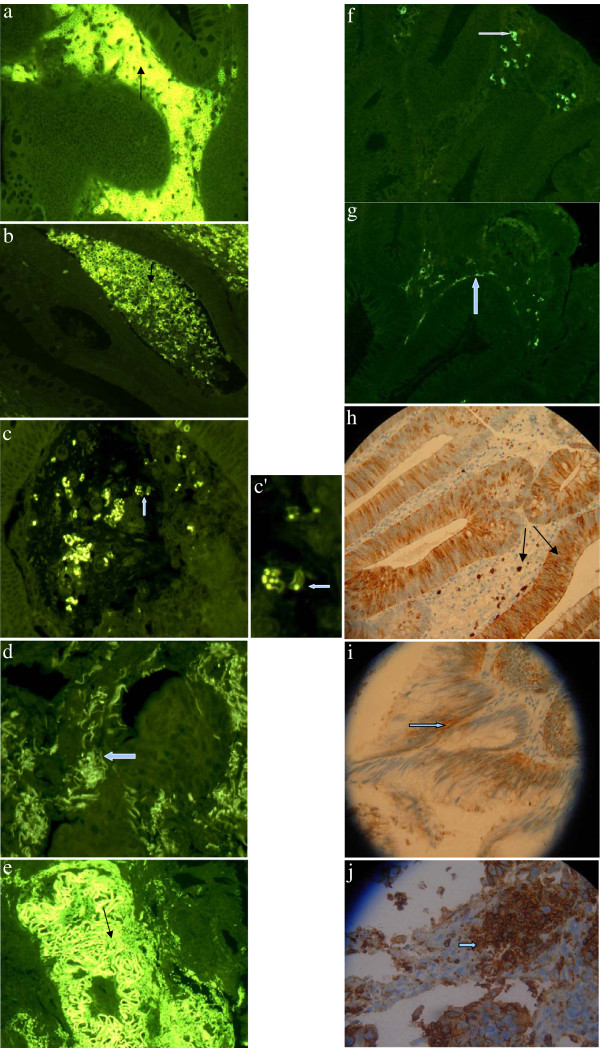
**Morphological distribution of μ chains in a pre-malignant polyp revealed by means of indirect immunofluorescence (magnification 40x).** The IPC structures were regularly delineated and, unlike conventional plasma cells, had a low cytoplasmic/nuclear ratio.

The staining of the pre-malignant polyp biopsies showed a striking invasion of small peri-glandular IgM-positive cells with a low cytoplasmic/nuclear ratio in the hyperplastic intestinal mucosa (Figure 1a). However, there were morphological modifications in some zones, and the irregular distribution of anti-μ chain fluorescence seemed to indicate the initial disintegration of IPC membranes (Figure 1b) or an accumulation of fluorescence in individual IPCs (Figure 1c and c’).

### B cell surface phenotype analysis of μ chain-positive cells identified a subset of λ and κ chain positive, CD79+/CD20-/CD21-/CD23- IPCs in pre-malignant polyps

B cell-specific surface molecules were examined in the four polyp samples, all of which were positive for CD79 and κ and λ chains. This finding was confirmed by IIF. All of the samples were negative for the three other specific markers of the B2 subset: CD20 (a mature B cell-specific molecule), CD23 and CD21. Morphology and phenotype analyses indicated that these cells were similar to B1 cells. They were also present as discrete foci in ten normal small intestinal, colonic and rectal biopsy specimens, which suggests they contribute to the physiological intestinal cell population.

### Anti-μ chain marker localisation in colorectal adenocarcinomas identified B cell membrane fragments

Six of the ten adenocarcinoma samples showed a loss in the integrity of lymphocytic cell membranes, and the fluorescent anti-μ chain marker was either distributed across membrane segments (Figure 1d) or concentrated on irregular membranous structures. These newly-formed membranes isolated small tissue portions within the tumoral context (Figure 1e). Morphologically, the fragments of membrane seemed to come from small B1-like cells with a low cytoplasmic/nuclear ratio, which had lost their nuclei and were distributed around the gland-like structures. Within the same biopsy stainings, fluorescent anti-μ antibodies also revealed very thick, irregular and stratified articulated membranes that completely enclosed portions of neoplastic tissue. Their peripheral zones showed persistent membrane fragments whose morphology indicated they came from B1-like cells. The remaining four samples contained IgM-positive cells with irregular nuclei that were sporadically distributed and had a peri-glandular localisation (Figure 1f). These cells were not detected in the normal control tissue. The samples also showed the progressive dissolution of lymphocytic membranes. The fluorescent anti-μ chain marker was distributed around glandular structures in a fragmentary manner (Figure 1g*).* No IPC membrane remodelling was detected in these samples.

### CD15 marker distribution within the pre-malignant polyps indicated epithelial and inflammatory cell integrity

In the pre-malignant polyps, the CD15 marker was localised on the surface of glandular cells or on inflammatory cells sporadically infiltrating the peri-glandular zone (Figure 1h). The intercellular matrix clearly showed epithelial cells with undamaged membranes.

### CD15 marker distribution within the adenocarcinomas indicated epithelial cell membrane disintegration

The CD15 marker on the adenocarcinoma stainings indicated the disintegration of both inflammatory and epithelial cell membranes (Figure 1i). The integrity of the cells and the intercellular space was lost, and there was an irregular accumulation of the CD15 marker in circumscribed tissue areas (Figure 1j).

## Discussion

The findings of this morphological study indicate that IPCs and CD15+ cells were present in the neoplastic foci, and participated in the local transformations. The μ chain-positive IPCs in the premalignancy polyps were small CD79+/CD20-/CD21-/CD23- lymphocytes with a low cytoplasmic/nuclear ratio. They therefore had a surface phenotype that is unrelated to mature B cells and seemed to represent a distinct subset of B1-like IPCs. These cells had the same morphological aspects and membrane markers of IPCs found in 42.7% of the IBD patients involved in our previous study [8]. However, unlike the findings in the IBD samples, there were circumscribed areas in which anti-μ chain fluorescence was irregularly distributed, which may indicate initial membrane disintegration. There was also an accumulation of fluorescence on the membrane surface of individual IPCs that suggests probable reactivity against tissue component. In patients with IBD, the CD15 marker is regularly expressed on epithelial and inflammatory cells, and the integrity of the cell membranes is maintained, and this was observed in the polyp samples despite the presence of epithelial hyperplasia. On the contrary, the CD15 marker revealed epithelial and inflammatory cell fragments indicating the disintegration of both in all of the adenocarcinoma specimens. The findings of an increasing number of studies suggest that stromal cells play a significant role in the process of tumour invasion [10]. The CD15-positive fragments occupied the intercellular matrix, thus suggesting that the alteration may have caused the subversion of normal membrane disposition. *In vitro* and *in vivo* models have shown that anti-tumour antibodies mediate tumour invasion and metastasis by processes that include extracellular matrix degradation and angiogenesis [11]. Matrix metalloproteinase 2 (produced particularly by inflammatory cells) [12] and matrix metalloproteinase 7 (encoded by epithelial cells) are both involved in the degradation of extracellular matrix components, and have been related to carcinogenesis and metastasis in patients with colorectal cancer [13]. The increased activity of matrix metalloproteinases in adenomas appears to be a biomarker of early tumorigenesis [14]. In six of the ten adenocarcinoma biopsy stainings, the μ chains had lost their normal disposition but could be recognised on membrane segments. Morphologically, it seemed that the μ chain-positive segments came from B1-like cells. In previous studies [8,9], we have found B1-like cells in normal and inflammatory intestinal tissue, and in polyps. These cells therefore live on intestinal mucosa, and their number may increase during the inflammatory process. Neoplastic transformation changes their morphology somewhat in polyps but markedly in adenocarcinomas, to the point that they lose their structural integrity. These findings suggest that the immunoglobulins detected in the neoplastic lesions did not come from circulating cells or a remote control mechanism. It therefore seems that there is no deposition of immunoglobulins in adenocarcinomas [15], but a loss of the integrity of resident cells expressing IgM. The same six adenocarcinoma biopsy stainings showed membrane remodelling and the overexpression of μ chains and CD15 receptors, which was identified as atypical membrane remodelling arising from isolated tissue portions. This was not found in the previously evaluated sections of normal or inflamed intestinal tissue and polyps. On the basis of the images, we believe that these tissue islands are generated by a membrane fusion mechanism, and that the μ chains and CD15 marker acquired in this remodelling process give the newly-formed membranes adhesive properties. As the CD15 receptor mediates adhesion to vascular walls [16] and antiphosphatidylcholine antibodies are directed against components of normal cell membranes [17], we hypothesise that the concentration of these receptors in restricted tissue areas can increase tissue adhesion in vessel walls and cell membranes, and thus induce metastases. Studies of the associations between cell surface molecules and the metastatic behaviour of tumour cells in humans have shown that the activity of the collagenolytic enzymes produced and secreted by tumour tissues has no correlation with metastatic potential, but that the expression of a high-molecular-weight mucin-like sialoglycoprotein positively correlates with progression to metastatic phenotypes [18,19]. Furthermore, studies of the induction of metastases in B cell knockout and B cell-depleted mice have shown that there a reduction in tumour growth and metastases when B cells are absent or impaired [11]. However, the remaining four of our tumour samples showed the infiltration of IgM-positive cells with irregular nuclei. These cells infiltrated the tissue in a sporadic manner and were often fragmented (which complicates their additional characterisation), but their microscopic appearance is very particular and identified the same cells previously observed in 35.7% of an IBD population [9]. The cells had lost their structural integrity, but there was no membrane remodelling. The fluorescent marker of immunoglobulin was scarce, had a peri-glandular disposition and affected the partial demarcation of tumoral tissue. We therefore suggest that the presence of different types of IPCs in tumoral transformation foci can influence the behaviour of malignant tumours, and the number and quality of the receptors on the tumour surface. It has been previously shown that the size of primary tumours with synchronous hepatic metastases is not significantly different from that of non metastatic tumours, which suggests that certain tumours inherently have greater metastatic potential depending on a variety of specific biochemical properties other than those of poorly metastatic cells [20]. Our morphological findings show the existence of a matrix degradation and cell remodelling mechanism in colorectal adenocarcinomas that involves both IPCs and epithelial cells. The remodelling and concentration of μ chains in established cancer areas are extremely relevant factors in the presence of a particular B1-like cell type. Experiments have shown that B1 cells are the main source of natural antibodies and that approximately 10-15% of peritoneal B1 cells are specific for the membrane phospholipid phosphatidylcholine. Phosphatidylcholine is the predominant type of phospholipid in malignant and control tissues, and its high levels in colon tumours is due to its decreased turnover and increased expression [21]. We hypothesise that IPCs play a role in the pathogenesis of colorectal tumours in the presence of alterations in tissue lipid composition, although further studies are necessary to clarify this and whether our model can be used for other forms of adenocarcinoma.

## Conclusion

In conclusion the findings of this preliminary morphological study suggest the presence of membrane disintegration and remodelling mechanisms in the tumours. The newly-formed membranes expressed high concentrations of inflammatory cell receptors that can confer adhesive properties.

## Abbreviations

IBD: Inflammatory bowel disease; IPC: Immunoglobulin-producing cell; IIF: Indirect immunofluorescence; UC: Ulcerative colitis; CD: Crohn’s disease; mAb: Murine monoclonal antibody; DAB: 3 3’-diaminobenzidine tetrahydrochloride; MMP: Matrix metalloproteinase.

## Competing interests

The authors declare that they have no competing interests.

## Authors' contributions

CD and SG carried out the Immunofluorescence and Immunoperoxidase method and drafted the manuscript. EM, AC, SS and SB participated in the coordination of the study. CD, FA, PSP participated in the design and coordination of the study and helped to draft the manuscript. SB participated in the design of the study and performed the statistical analysis. PLA conceived of the study, and participated in its design and coordination. All authors read and approved the final manuscript.

## Pre-publication history

The pre-publication history for this paper can be accessed here:

http://www.biomedcentral.com/1472-6890/13/8/prepub
